# *Talaromyces (Penicillium) marneffei* infection in non-HIV-infected patients

**DOI:** 10.1038/emi.2016.18

**Published:** 2016-03-09

**Authors:** Jasper FW Chan, Susanna KP Lau, Kwok-Yung Yuen, Patrick CY Woo

**Affiliations:** 1 State Key Laboratory of Emerging Infectious Diseases, The University of Hong Kong, Hong Kong, China; 2 Department of Microbiology, The University of Hong Kong, Hong Kong, China; 3 Research Centre of Infection and Immunology, The University of Hong Kong, Hong Kong, China; 4 Carol Yu Centre for Infection, The University of Hong Kong, Hong Kong, China

## Abstract

*Talaromyces (Penicillium) marneffei* is an important pathogenic thermally dimorphic fungus causing systemic mycosis in Southeast Asia. The clinical significance of *T. marneffei* became evident when the human immunodeficiency virus (HIV)/acquired immunodeficiency syndrome epidemic arrived in Southeast Asia in 1988. Subsequently, a decline in the incidence of *T. marneffei* infection among HIV-infected patients was seen in regions with access to highly active antiretroviral therapy and other control measures for HIV. Since the 1990s, an increasing number of *T. marneffei* infections have been reported among non-HIV-infected patients with impaired cell-mediated immunity. Their comorbidities included primary adult-onset immunodeficiency due to anti-interferon-gamma autoantibodies and secondary immunosuppressive conditions including other autoimmune diseases, solid organ and hematopoietic stem cell transplantations, T-lymphocyte-depleting immunsuppressive drugs and novel anti-cancer targeted therapies such as anti-CD20 monoclonal antibodies and kinase inhibitors. Moreover, improved immunological diagnostics identified more primary immunodeficiency syndromes associated with *T. marneffei* infection in children. The higher case-fatality rate of *T. marneffei* infection in non-HIV-infected than HIV-infected patients might be related to delayed diagnosis due to the lack of clinical suspicion. Correction of the underlying immune defects and early use of antifungals are important treatment strategies. Clinicians should be familiar with the changing epidemiology and clinical management of *T. marneffei* infection among non-HIV-infected patients.

## INTRODUCTION

**Figure 1:**
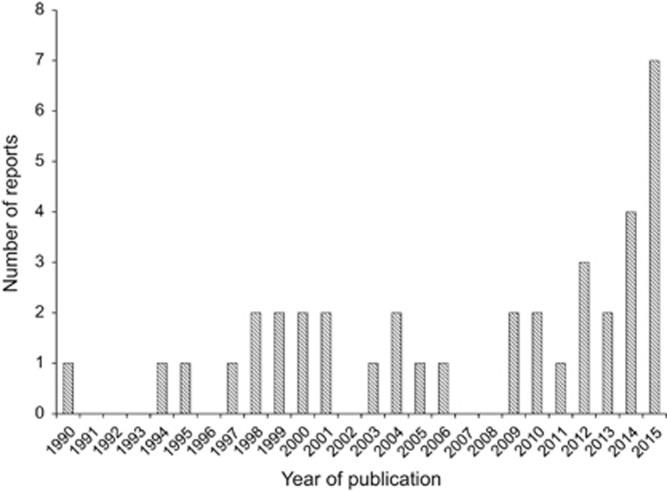
The number of reports of *Talaromyces marneffei* infection in non-HIV-infected adult patients described in the English-language literature between 1 January 1990 and 1 October 2015. Reports involving patients with uncertain human immunodeficiency virus infection status were excluded.

*Talaromyces (Penicillium) marneffei* is an important pathogenic thermally dimorphic fungus causing systemic mycosis in Southeast Asia.^1, 2, 3^ *T. marneffei* is a member of the family *Trichocomaceae*, order *Eurotiales*, class *Eurotiomycetes*, division *Ascomycota.* It is the only member in the *Talaromyces* genus which is considered to be an important human pathogen. *T. marneffei* infection is endemic in tropical regions, especially Thailand, Vietnam, northeastern India, Southern China, Hong Kong, Taiwan, Laos, Malaysia, Myanmar, Cambodia and Laos.^1^ The fungus was first isolated from the hepatic lesions of a bamboo rat (*Rhizomys sinensis*) which died spontaneously from the infection in 1956.^4^ Subsequent studies showed that bamboo rats (*Rhizomys* sp. and *Cannomys* sp.) and soil from their burrows were important enzootic and environmental reservoirs of *T. marneffei*, respectively.^4, 5, 6, 7^ The prevalence of *T. marneffei* infection in these susceptible animal species varies widely across Southeast Asia. Historically, *T. marneffei* infection in human has been considered to be exclusively associated with acquired immunodeficiency syndrome (AIDS) caused by human immunodeficiency virus (HIV) infection.^1, 8^ In some regions such as Hong Kong and southern China, *T. marneffei* infection has long been considered as one of the top three AIDS-defining opportunistic infections, alongside tuberculosis and cryptococcosis.^2, 9^ In recent years, improved treatment of HIV infection with highly active antiretroviral therapy and control of the HIV/AIDS epidemic with other measures have led to a change in the epidemiology of *T. marneffei* infection, with an increasing number and proportion of cases being reported in non-HIV-infected patients who had other immunocompromising conditions (Figure 1). *T. marneffei* infection in non-HIV-infected children has been discussed elsewhere.^10^ In this article, we thoroughly reviewed the epidemiological and clinical characteristics of *T. marneffei* infection among non-HIV-infected adult patients, and discussed on the specific management strategies for each at-risk group.

## THE CHANGING EPIDEMIOLOGY OF *T. MARNEFFEI* INFECTION

**Figure 2:**
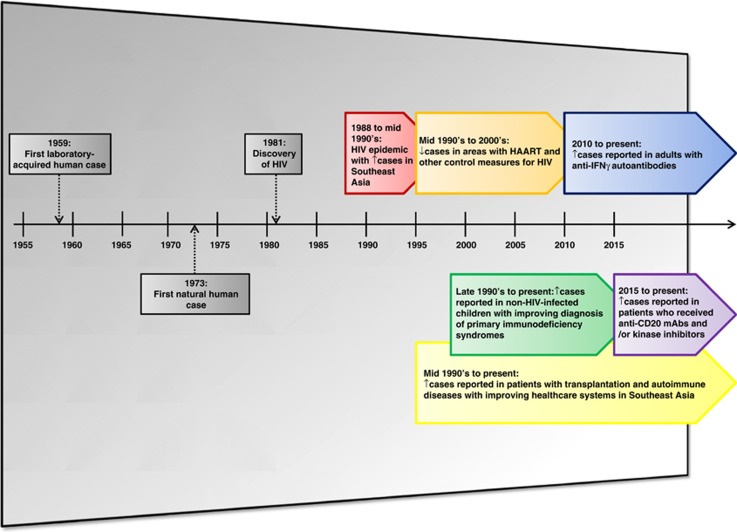
Major milestones in the changing epidemiology of *Talaromyces marneffei* infection. HAART, highly active antiretroviral therapy; HIV, human immunodeficiency virus; IFN-γ, interferon-gamma; mAb, monoclonal antibodies.

The first human case of *T. marneffei* infection occurred as a laboratory-acquired infection in 1959^11^ (Figure 2). A laboratory researcher accidentally inoculated the fungus into his own finger while performing experiments on mice and caused a localized small nodule at the inoculation site.^11^ The first natural human case of infection was reported in 1973 and involved an American minister with Hodgkin's disease who resided in Southeast Asia.^12^ Over the next 10 to 15 years, a few more sporadic cases were reported in Thailand, Hong Kong and southern China.^13, 14, 15, 16, 17, 18, 19, 20, 21, 22^ The HIV status of most of these patients was not known as the virus was not discovered until 1981 and laboratory diagnostics for HIV infection was not readily available in Southeast Asia in the early 1980s. The incidence rate of *T. marneffei* infection markedly increased after the HIV/AIDS epidemic arrived in Southeast Asia in 1988.^1^ *T. marneffei* infection was reported not only among HIV-infected patients residing in endemic areas, but also in HIV-infected patients who had traveled to these endemic areas.^1^

The economic boom in Southeast Asian countries since the 1990s was accompanied by an improvement in their healthcare infrastructures. These included better control of HIV infection and improved diagnosis of non-AIDS conditions associated with impaired cell-mediated immunity. The availability of highly active antiretroviral therapy and other control measures for the HIV/AIDS epidemic led to a decrease in the incidence rate of HIV-associated *T. marneffei* infection.^1^ On the other hand, *T. marneffei* infection was increasingly reported in different groups of patients with primary or secondary immunocompromising conditions (Figure 2). The use of potent immunosuppressive drugs in patients with transplantation and autoimmune diseases was associated with an increased incidence of *T. marneffei* infection among these non-HIV-infected patients since the mid 1990s. Improved genetic testing for various primary immunodeficiency syndromes led to the recognition of more cases of *T. marneffei* infection in non-HIV-infected children.^10, 23^ The recently identified association between *T. marneffei* infection and the adult-onset immunodeficiency syndrome caused by anti-interferon-gamma (anti-IFN-γ) autoantibodies helped to explain many previous cases of *T. marneffei* infection among non-HIV-infected adult Asian patients who had no other comorbidities.^24^ Recently, *T. marneffei* infection was also observed in non-HIV-infected hematology patients who were treated with novel targeted therapies including anti-CD20 monoclonal antibodies and kinase inhibitors.^25, 26^

## OVERVIEW OF THE IMMUNOLOGICAL BASIS AND CLINICAL CHARACTERISTICS OF *T. MARNEFFEI* INFECTION IN NON-HIV-INFECTED ADULT PATIENTS

*T. marneffei* proliferates in macrophages and disseminates via the reticuloendotheial system.^1^ Clinically, the infection is characterized by fungal invasion of multiple body organ systems, especially blood, bone marrow, skin, lungs and reticuloendothelial tissues.^1^ Similar to other intracellular pathogens, the activation of macrophages by T-lymphocyte-derived cytokines, especially those of the Th1 response such as interleukin (IL)-12, IFN-γ and tumor necrosis factor (TNF)-α, is important for host defense against *T. marneffei* infection.^27^ This is supported by the observation that *T. marneffei* infection in nude or T-lymphocyte-depleted mice was invariably fatal, whereas the fungus could be cleared within three weeks in healthy mice.^28, 29^ A polarized Th1 response prevented immunoevasion by *T. marneffei* in parasitized mononuclear phagocytes and stimulated macrophage killing of intracellular *T. marneffei* via the L-arginine-dependent nitric oxide pathway.^30, 31^ Furthermore, granuloma formation, which is important for containment of the fungus, was found in wild-type mice but not in IFN-γ-knocked out mice.^27^ These evidences suggest that patients with defective cell-mediated immunity may be at risk of developing *T. marneffei* infection.

The severity of the infection varies among patients with different degrees of immunosuppression. In HIV-infected patients, *T. marneffei* infection is often disseminated and involves multiple organs.^1, 2^ In non-HIV-infected patients, the infection may be focal or disseminated, depending on the underlying immunocompromising condition and the timing of diagnosis. In a retrospective cohort study involving 116 HIV-infected and 34 non-HIV-infected patients with *T. marneffei* infection in Thailand, it was found that the non-HIV-infected patients were significantly older, less likely to have fever, splenomegaly and umbilicated skin lesions, and more likely to have Sweet's syndrome and osteoarticular lesions.^32^ The non-HIV-infected patients also had higher leukocyte, CD4 lymphocyte and platelet counts, and lower alanine transaminase level and blood culture-positive rate.^32^

To further analyze the clinical characteristics of non-HIV-infected patients with *T. marneffei* infection, we reviewed the reports of non-HIV-related *T. marneffei* infection in the English-language literature found in a PubMed search using the key words ‘*Talaromyces*', ‘*Penicillium*', ‘*marneffei*' and ‘penicilliosis' on 1 October 2015. Reports involving patients with uncertain HIV status were excluded. A total of 119 patients with detailed clinical information were identified (Table 1). There were 65 males and 54 females. Their median age was 42.8 years (range, 22 to 79 years). Common clinical features of *T. marneffei* infection among these non-HIV-infected patients included fever, malaise, weight loss, skin and soft tissue lesions, hepatosplenomegaly, lymphadenopathy, cough and dyspnea (Table 2). Some patients also had osteoarticular involvement and abdominal symptoms such as abdominal pain and diarrhea due to mesenteric lymphadenopathy or terminal ileitis mimicking Crohn's disease. Less common clinical features included tracheomediastinal fistula and neurological manifestations such as seizure and confusion due to the presence of intracranial lesions. Laboratory investigations often revealed leukocytosis or leukopenia, anemia, thrombocytosis, deranged liver function test results and elevated inflammatory marker levels including those of C-reactive protein and erythrocyte sedimentation rate. Patients with pulmonary involvement exhibited various chest X-ray abnormalities, including uni- or multi-lobar consolidations, cavities, interstitial infiltrates, pleural effusion, pericardial effusion and enlarged hilar shadow due to mediastinal and hilar lymphadenopathies.

Overall, 33/119 (27.7%) non-HIV-infected patients with *T. marneffei* infection died despite most of them having received antifungal treatment with anti-*T. marneffei* activity, such as amphotericin B, itraconazole and voriconazole (Table 2). This case-fatality rate was similar to that of non-HIV-infected patients with *T. marneffei* infection in another study, in which 10/34 (29.4%) died.^32^ Both of these rates were higher than that of HIV-infected patients (24/116, 20.7%) in the same cohort, and might reflect delayed diagnosis of *T. marneffei* infection among non-HIV-infected patients due to the lack of clinical suspicion in the early stage. Notably, many of the non-HIV-infected patients with *T. marneffei* infection were initially misdiagnosed and empirically treated as tuberculosis because both infections are endemic in Southeast Asia, have similar predisposing factors and overlapping clinical manifestations (Table 2). The diagnosis of *T. marneffei* infection in these patients was often established weeks to months later when the clinical condition failed to improve to empirical anti-tuberculosis treatment. Moreover, some of the patients residing in non-endemic regions only became symptomatic months to years after returning from endemic areas.^56, 59^ These factors likely led to delayed commencement of appropriate antifungal treatment. Familiarity with the non-AIDS conditions associated with *T. marneffei* infection would facilitate clinicians to improve the clinical management of the infection among these at-risk patients.

## SPECIFIC CHARACTERISTICS OF NON-AIDS CONDITIONS ASSOCIATED WITH *T. MARNEFFEI* INFECTION

### Anti-IFN-γ autoantibodies

Immunodeficiency due to anti-IFN-γ autoantibodies is an emerging adult-onset immunodeficiency syndrome first described in 2004.^68, 69^ The affected patients have high-titer serum neutralizing anti-IFN-γ autoantibodies that inhibit STAT1 phosphorylation and IL-12 production, leading to a severely compromised Th1 response.^70^ As a result, these patients develop recurrent, severe and/or disseminated opportunistic infections caused by various intracellular pathogens.^24, 70^ Over the past decade, this condition has been increasingly reported among adult Asian patients, including Filipino, Thai, Vietnamese, Japanese and Chinese residents in Hong Kong, Taiwan and mainland China.^24, 57, 70, 71, 72, 73, 74, 75^ This ethnic predilection is likely related to genetic predispositions among Asians. Recently, the association between anti-IFN-γ autoantibodies and HLA class II alleles, including HLA-DR*15:02/16:02 and HLA-DQ*05:01/05:02, was reported.^72, 76^

As the initial reports mostly involved patients residing in areas non-endemic of *T. marneffei*, only non-tuberculous mycobacteriosis was recognized as an important opportunistic pathogen in these patients.^68, 69, 77^ It was not until 2010 when the association between anti-IFN-γ autoantibodies and *T. marneffei* infection was described among eight Chinese patients living in Hong Kong.^24^ The susceptibility of patients with anti-IFN-γ autoantibodies to other intracellular pathogens including non-typhoidal *Salmonella* sp., *Burkholderia* sp., varicella zoster virus and less commonly, *Histoplasma capsulatum* and *Cryptococcus neoformans*, was also recognized subsequently.^24, 70^ *T. marneffei* infections in patients with anti-IFN-γ autoantibodies usually manifest as fever of unknown origin, cervical lymphadenitis and/or mild symptomatic infection with positive serology.^24^ Occasionally, *T. marneffei* infection or non-tuberculous mycobacteriosis might precipitate the development of reactive dermatoses such as Sweet's syndrome, erythema nodosum, exanthematous pustulosis and pustular psoriasis, or cause direct infective cutaneous lesions in these patients.^51^

The treatment of *T. marneffei* infection in patients with anti-IFN-γ autoantibodies comprises of both effective antifungal therapy and immunomodulation to control the underlying immunological defect. These patients often responded poorly or developed recurrent infections when they were treated with antifungal therapy alone. The most effective immunomodulating treatment available currently is rituximab, an anti-CD20 antibody which targets B lymphocytes to reduce the production of serum-neutralizing anti-IFN-γ autoantibodies.^78, 79^ However, since rituximab has recently been identified as a potential risk factor for *T. marneffei* infection, a delicate balance to minimize the level of anti-IFN-γ autoantibodies, while not over-suppressing the immune system, needs to be established.^25^ The dosing regimen and time intervals of administering rituximab in patients with anti-IFN-γ autoantibodies should be further evaluated in larger clinical trials.

### Other autoimmune diseases

*T. marneffei* infection has been reported in at least 15 patients with various other autoimmune diseases, including systemic lupus erythematosus (SLE), mixed connective tissue disease, Sjögren's syndrome, primary biliary cirrhosis, primary immune (idiopathic) thrombocytopenia and autoimmune hemolytic anemia.^32, 34, 35, 36, 37, 40, 41, 43, 45, 53, 54, 80^ Although the immunological defects of these autoimmune diseases were variable, the predisposing factors for *T. marneffei* infection could be broadly classified into treatment-related and disease-related. In patients with organ- or tissue-specific conditions, such as Sjögren's syndrome, primary biliary cirrhosis, primary immune thrombocytopenia and autoimmune hemolytic anemia, the degree of systemic immunosuppression was usually not severe. Therefore, *T. marneffei* infection usually occurred when these patients received high-dose or prolonged treatment with T-lymphocyte-depleting drugs, including corticosteroids, cyclosporine, azathioprine, tacrolimus and mycophenolate mofetil. Treatment of *T. marneffei* in these patients usually required a reduction of immunosuppressive drugs together with antifungal therapy. In patients with SLE or mixed connective tissue disease who had more severely compromised cell-mediated immunity, *T. marneffei* infection might occur even with less immunosuppressive therapy. In patients with active SLE, marked lymphopenia may occur in the absence of immunosuppressive therapy and is an important risk factor for not only *T. marneffei* infection, but also opportunistic infections caused by other intracellular organisms such as *Mycobacterium tuberculosis*, non-tuberculous mycobacteria, *Nocardia* sp., *Rhodococcus* sp., *Burkholderia pseudomallei*, *C. neoformans*, *Pneumocytis jirovecii*, *Toxoplasma gondii* and herpesviruses.^81, 82^ The management of *T. marneffei* infection in these patients thus requires both effective antifungal agents and careful titration of immunosuppressive therapy to control the underlying lupus activity.

### Organ transplantation

*T. marneffei* infection has been occasionally reported in solid organ transplantation and hematopoietic stem cell transplantation (HSCT) recipients. Most of these patients developed *T. marneffei* infection when escalated doses of immunosuppressive drugs were used to treat graft rejection. *T. marneffei* infection was otherwise uncommon among transplantation recipients who had minimal maintenance anti-rejection therapy. The most common type of transplantation associated with *T. marneffei* infection was renal transplantation, with at least 12 cases having been reported.^22, 36, 38, 41, 44, 45, 46, 52, 56, 83^ *T. marneffei* infection has also been uncommonly reported in liver transplantation, lung transplantation and HSCT recipients on multiple T-lymphocyte-depleting immunosuppressive drugs.^47, 66, 80, 84^

Due to the small number of cases, it is difficult to determine the reasons for the apparently higher incidence of *T. marneffei* infection among renal than other solid organ transplantation and HSCT recipients. Possible reasons include the earlier adoption and higher annual number of renal transplantation than the other types of transplantation in *T. marneffei-*endemic areas, and the different antifungal prophylaxis regimens used in these transplantation recipients. For example, in Hong Kong, the first cadaveric and living-related donor renal transplantations were performed in 1969 and 1980, respectively.^85^ The first HSCT was performed in 1990, and the first liver and lung transplantations were performed in 1991 and 1995, respectively.^86, 87, 88^ Furthermore, renal transplantation has consistently remained as one of the most common types of solid organ transplantation performed each year in Hong Kong over the past few decades. While HSCT recipients are generally considered to have more severe immunosuppression and thus higher risk of developing invasive fungal infections than solid organ transplantation recipients, they tend to receive more potent and prolonged antifungal prophylaxis and/or empirical antifungal treatment with activities against *T. marneffei*, such as itraconazole, voriconazole, posaconazole and amphotericin B.^89, 90, 91, 92, 93^ In contrast, fluconazole and nystatin are commonly used as antifungal prophylaxis in patients with solid organ transplantations including renal transplantation, and thus may not be effective against *T. marneffei* infection.

### Hematological malignancies and novel anti-cancer targeted therapies

In addition to a HSCT recipient with IgA myeloma, *T. marneffei* infection has also been reported in a few adult patients with hematological malignancies or proliferative diseases including non-Hodgkin's lymphoma, Waldenström's macroglobulinemia and Langerhans cell histiocytosis.^32, 42, 43, 47, 48, 67, 80^ The incidence of *T. marneffei* infection in this group of patients has remained low in the past decades. Recently, however, we were alerted by four unprecedented cases of disseminated *T. marneffei* infection among hematology patients who received novel targeted therapies including anti-CD20 monoclonal antibodies and kinase inhibitors.^25, 26^

Rituximab and obinutuzumab are types I and II anti-CD20 monoclonal antibodies, respectively, that predominantly target B lymphocytes. In contrast to Th1 response, the role of B-lymphocyte-mediated humoral response in *T. marneffei* infection is not well-defined. Patients with B-lymphocyte dysfunction may have impaired production of neutralizing antibodies against key virulence factors of *T. marneffei* identified in genome sequencing, proteome profiling and other downstream studies.^94, 95, 96, 97, 98, 99, 100, 101, 102, 103, 104, 105, 106, 107, 108^ Treatment with rituximab induces long-lasting B-lymphocyte-depleting effects. B-lymphocyte reconstitution, characterized by the expansion of functionally immature B lymphocytes and decreased memory B lymphocytes, may take more than one year after treatment completion.^109^ During this period, latent infections such as hepatitis B or even *T. marneffei* infection may become reactivated.^110^ The newer obinutuzumab is even more potent than rituximab in depleting B lymphocytes.^111^ Therefore, *T. marneffei* infection should be considered not only in patients who are receiving, but also those who have already completed treatment with anti-CD20 monoclonal antibodies, when they develop compatible clinical features.

Kinase inhibitors such as ruxolitinib and sorafenib have been increasingly used to treat hematological and solid organ malignancies and/or benign conditions such as psoriasis and alopecia acreata in recent years. Ruxolitinib is a selective Janus kinase (JAK) 1 and 2 inhibitor that interferes with the signal transduction for types I and II cytokines including IFN-γ and the JAK-STAT pathway.^112^ Besides *T. marneffei* infection, opportunistic infections and reactivation due to other intracellular organisms, including *M. tuberculosis*, *C. neoformans*, herpes simplex virus and hepatitis B virus, have also been reported in patients who received treatment with ruxolitinib.^113, 114, 115, 116^ Sorafenib is a multi-kinase inhibitor that exhibits various immunomodulatory effects, including impairment of T-lymphocyte proliferation, IFN-γ production, natural killer cell activity, dendritic cell function and pro-inflammatory cytokine secretion.^117, 118, 119^ The use of sorafenib has been associated with reactivation of latent tuberculosis.^120^ With the expanding list of targeted therapies becoming available in the market and being used in endemic regions of *T. marneffei*, it would be important for clinicians to maintain a high index of suspicion and possibly perform serial serological surveillance for *T. marneffei* infection in patients who have received these agents to avoid a delay in diagnosis and treatment.^121^

### Other non-AIDS conditions

Sporadic cases of *T. marneffei* infection have been reported in a few other non-HIV-infected patients. Their underlying conditions included idiopathic CD4+ thrombocytopenia, Job's syndrome, diabetes mellitus, splenectomy and colonic, breast and buccal cancers.^32, 43, 45, 49, 50, 54, 62, 65, 67^ However, it is difficult to assess the exact role of these conditions in *T. marneffei* infection because of the limited number of cases. Interestingly, an increasing number of non-HIV-infected Asian patients with *T. marneffei* infection who were previously considered to have no underlying comorbidities were subsequently found to be positive for anti-IFN-γ autoantibodies.^32^ Advances in immunological diagnostics may help to identify more novel immunodeficiency syndromes and their association with *T. marneffei* infection in the future.

## CONCLUDING REMARKS

The epidemiology of *T. marneffei* infection has changed significantly in the past three decades. It is now widely recognized that the infection is not limited to HIV-infected patients. Looking ahead, more cases of *T. marneffei* infection are likely to be reported in the future because of several reasons. First, improvement in the healthcare systems of developing countries in Southeast Asia, such as mainland China, Thailand and Vietnam, will likely lead to an enlarging population of non-HIV-infected patients at risk of the infection, including transplantation recipients and cancer patients on targeted therapies. The discovery of novel immunodeficiency syndromes in children and adults will continue to identify more at-risk patient groups. The availability of new diagnostic and typing methods will facilitate the detection and molecular characterization of the *T. marneffei* strains infecting the patients in these areas.^9, 121, 122, 123, 124, 125, 126^ Finally, further studies to address key questions regarding the use of prospective surveillance and optimal treatment strategies will become feasible with this continuously expanding population of non-HIV-infected patients with *T. marneffei* infection.

## Figures and Tables

**Table 1:** *Talaromyces marneffei* infection in non-HIV-infected adult patients^a^

**Sex (number)**	**Age (year)**	**Comorbidities**	**Notable findings**	**Outcome (treatment)**	**Ref.**
F (1)	51	Old pTB	Possibly the first report of *T. marneffei* infection in a patient with HIV status known to be negative	Recovered (AmpB and 5-fluorocytosine)	Chan *et al*^22^
M (1) and F (2)	23–58	Old pTB	Rare report of osteoarticular involvement in non-HIV-infected patients with *T. marneffei* infection	2/3 recovered (AmpB and/or itraconazole); 1 died.	Louthrenoo *et al*^33^
F (1)	29	SLE on prednisolone	Rare report of *T. marneffei* infection in a patient with SLE, treated as TB for 3 weeks	Recovered (AmpB and itraconazole)	Lo *et al*^34^
F (1)	65	SLE on immunosuppressants (prednisolone and azathioprine), hypertension	Rare report of *T. marneffei* in a patient with SLE	Died	Lam *et al*^35^
M (1) and F (1)	43 and 35	ITP, renal transplantation, immunosuppressants (corticosteroid, azathioprine)	First report of indigenous *T. marneffei* infection in Taiwan	Died (AmpB)	Hung *et al*^36^
F (1)	45	Sjögren's syndrome on prednisolone	Rare report of reactive hemophagocytosis associated with *T. marneffei* infection	Improved with antifungal treatment (AmpB) but later died of nosocomial catheter-related staphylococcal blood stream infection	Chim *et al*^37^
M (1)	33	Renal transplantation on immunosuppressants (prednisolone, azathioprine, tacrolimus and cyclosporine)	Rare report of intestinal *T. marneffei* infection	Died	Ko *et al*^38^
F (1)	48	None	First report of *T. marneffei* infection in a non-HIV-infected patient in Malaysia	Recovered (AmpB and itraconazole)	Saadiah *et al*^39^
F (1)	30	MCTD	Rare report of osteomyelitis; treated as TB for >6 months	Recovered	Pun *et al*^40^
M (4) and F (2)	33–58	SLE, ITP, renal transplantation, immunosuppressants (corticosteroid, cyclosporine, azathioprine, tacrolimus)	First report of possible strain dissemination among Taiwan based on phenotypic and genotypic evidence	4/6 (66.7%) patients recovered, 2/6 (33.3%) died.	Hsueh *et al*^41^
M (1)	73	WM	First report of *T. marneffei* infection in a patient with WM (mixed fungemia due to *Candida tropicalis* and *T. marneffei*)	Died (AmpB)	Wong *et al*^42^
M (2) and F (5)	23–73	Hemic malignancy, Sjögren's syndrome on corticosteroid and cyclophosphamide, SLE on azathioprine, autoimmune hemolytic anemia on prednisolone, DM	First report comparing the clinical and laboratory features of *T. marneffei* infection in HIV-infected and non-HIV-infected patients	3/7 (42.9%) patients recovered, 4/7 (57.1%) died (AmpB and/or itraconazole)	Wong *et al*^43^
M (1)	47	Renal transplantation on immunosuppressants (tacrolimus and prednisolone)	Rare report of *T. marneffei* infection in a patient with renal transplantation	Recovered (AmpB and itraconazole)	Wang *et al*^44^
M (4) and F (3)	21–46	DM, SLE and renal transplantation	Rare report of *T. marneffei* infection in patients with SLE and renal transplantation	4/7 (57.1%) patients recovered, 3/7 (42.9%) died (AmpB and/or itraconazole)	Liyan *et al*^45^
M (1)	38	Cadaveric renal transplantation on immunosuppressants (tacrolimus, mycophenolate mofetil and prednisolone)	Rare report of *T. marneffei* infection in a patient with renal transplantation	Recovered (AmpB and itraconazole)	Chan *et al*^46^
M (1)	57	IgA myeloma with allogeneic HSCT on prednisolone	First report of *T. marneffei* infection in a patient with HSCT	Died of multi-organ failure (Amp B)	Lau *et al*^47^
M (2) and F (1)	35–45	Non-Hodgkin's lymphoma	Report on patients with pulmonary manifestations of *T. marneffei* infection	NA	Deesomchok *et al*^48^
M (1)	57	Idiopathic CD4+ lymphopenia	Rare report of *T. marneffei* infection in a patient with idiopathic CD4+ lymphopenia	Recovered (AmpB and itraconazole)	Beh*et al*^49^
M (1)	30	Job's syndrome	First report of *T. marneffei* infection in a patient with Job's syndrome	Died of respiratory failure	Ma *et al*^50^
M (4) and F (4)	39–87	Anti-IFN-γ autoantibodies	First report of *T. marneffei* infection in patients with anti-IFN-γ autoantibodies who also developed reactive dermatoses	Recovered (AmpB and/or itraconazole)	Tang *et al*^24^Chan *et al*^51^
F (1)	42	Cadaveric renal transplantation on immunosuppressants (prednisolone and tacrolimus)	Rare report of *T. marneffei* infection in renal transplant recipient	Recovered (AmphB and itraconzolae)	Lin *et al*^52^
F (1)	46	SLE on prednisolone	*T. marneffei* infection in SLE; treated as TB for 2 weeks	Recovered (AmpB and itraconazole)	Luo *et al*^53^
M (1) and F (1)	23 and 40	SLE on immunosuppressants (prednisolone, MMF, azathioprine and hydroxychloroquine), splenectomy	*T. marneffei* infection in SLE patients on immunosuppressants	Recovered (AmpB, voriconazole and/or itraconazole)	Chong *et al*^54^
M (1)	45	None	Endobronchial polypoid lesion with obstructive pneumonia	Recovered with surgery and antifungal treatment (AmpB and itraconazole)	Joosten *et al*^55^
M (1)	67	Cadaveric renal transplantation on immunosuppressants (tacrolimus, mycophenolate mofetil and prednisolone)	The patient developed fungemic peritonitis 2 months after returning from endemic regions	Recovered (Amp B and itraconazole)	Hart *et al*^56^
M (17) and F (17)	50–64	DM, lymphoma, colon cancer, SLE, MCTD, myasthenia gravis, immunosuppressants	Comparison between HIV- and non-HIV-infected patients with *T. marneffei* infection in northern Thailand	24/34 (70.6%) patients recovered, 10/34 (29.4%) died (AmpB and/or itraconazole)	Kawila *et al*^32^
F (2)	40 and 40	Anti-IFN-γ autoantibodies	Taiwanese patients with *T. marneffei* infection associated with auto-IFN-γ antibodies	Recovered (AmpB)	Lee *et al*^57^
M (1)	28	None	Rare report of tracheal stenosis and tracheomalacia	Recovered with surgery and antifungals (Amp B and itraconazole)	Qiu *et al*^58^
M (1)	79	COPD, old pTB	Developed chronic pulmonary *T. marneffei* infection years after returning from endemic regions	Recovered (AmpB and itraconazole)	De Monte *et al*^59^
M (1)	71	Immunosuppressants (prednisolone and azathioprine) for interstitial pneumonia	First case of *T. marneffei* infection non-HIV-infected patient in Japan	Recovered (itraconazole)	Furusawa *et al*^60^
F (1)	40	None	Rare report of osteolysis of sternum and clavicle	Recovered (AmpB)	Liu *et al*^61^
M (1)	38	Idiopathic CD4+ lymphopenia	Treated as TB for 11 months	Recovered (itraconazole and recombinant interleukin-2)	Xia *et al*^62^
F (1)	22	None	Rapid deterioration with multi-organ dysfunction and died on the day of admission; treated as TB for 5 months	Died of multi-organ failure	Jiang *et al*^63^
M (2)	32 and 37	None	Rare report of intracranial lesions with seizure and tracheomediatinal fistula; treated as TB for 2 months	Recovered (AmpB and itraconazole)	Ye *et al*^64^
M (4)	44–67	Anti-CD20 mAbs, kinase inhibitors, DM, WM, ITP, PBC, CLL, AML, myelofibrosis, splenectomy	First reports of *T. marneffei* infection associated with anti-CD20 and kinase inhibitors	Recovered (AmpB, itraconazole and/or voriconazole)	Chan *et al*^25^Tse *et al*^26^
M (1)	46	Left buccal cancer	Concomitant pulmonary tuberculosis	Recovered (AmpB and itraconazole)	Wang *et al*^65^
F (1)	41	Bilateral lung transplantation for cystic fibrosis, on immunosuppressants (prednisolone, mycophenolate and tacrolimus)	First case of *T. marneffei* infection in lung transplant recipient	Recovered (voriconazole)	Stathakis *et al*^66^
M (9) and F (5)	22–67	DM, corticosteroid, β-thalassemia, breast cancer and Langerhans cell histiocytosis	Rare report of osteoarticular lesions	8/14 (52.1%) patients recovered, 6/14 (42.9%) died	Qiu *et al*^67^

Abbreviations: AML, acute myeloid leukemia; AmpB, amphotericin B; CLL, chronic lymphocytic leukemia; COPD, chronic obstructive pulmonary disease; DM, diabetes mellitus; F, female; HIV, human immunodeficiency virus; HSCT, hematopoietic stem cell transplantation; IFN-γ, interferon gamma; ITP, idiopathic thrombocytopenic purpura; M, male; mAbs, monoclonal antibodies; MCTD, mixed connective tissue disease; MMF, mycophenolate mofetil; NA, not available; PBC, primary biliary cirrhosis; pTB, pulmonary tuberculosis; SLE, systemic lupus erythematosus; WM, Waldenström macroglobulinemia.

a Only reports in the English-language literature which provided the clinical details including the HIV status of the patients were included.

**Table 2:** Clinical and laboratory features of *Talaromyces marneffei* infections in non-HIV-infected adult patients^a^

**Clinical and laboratory features**	**Number of patients (%) (*n*=119)**
Fever	89 (74.8)
Malaise	48 (40.3)
Weight loss	34 (28.6)
Cough	50 (42.0)
Hemoptysis	4 (3.4)
Dyspnea	33 (27.7)
Hepatomegaly	23 (19.3)
Splenomegaly	19 (16.0)
Lymphadenopathy	50 (42.0)
Cutaneous or subcutaneous lesion	53 (44.5)
Osteomyelitis	25 (21.0)
Arthritis or arthralgia	16 (13.4)
Abdominal pain or diarrhea	15 (12.6)
Neurological manifestation	6 (5.0)
Leukocytosis	66 (55.5)
Leukopenia	13 (10.9)
Neutropenia	12 (10.1)
Lymphopenia	30 (25.2)
Anemia	47 (39.5)
Thrombocytosis	55 (46.2)
Thrombocytopenia	9 (7.6)
Fungemia	43 (36.1)
Misdiagnosed as tuberculosis	16 (13.4)
Death	33 (27.7)

Abbreviation: HIV, human immunodeficiency virus.

a All reports in Table 1 were included. Only data presented in the reports were included.

## References

[bib1] Vanittanakom N, Cooper CR Jr, Fisher MC et al. *Penicillium marneffei* infection and recent advances in the epidemiology and molecular biology aspects. Clin Microbiol Rev 2006; 19: 95–110. 16418525 10.1128/CMR.19.1.95-110.2006 PMC1360277

[bib2] Wong SS, Siau H, Yuen KY. *Penicilliosis marneffei*—West meets East. J Med Microbiol 1999; 48: 973–975. 10535639 10.1099/00222615-48-11-973

[bib3] Hu Y, Zhang J, Li X et al. *Penicillium marneffei* infection: an emerging disease in mainland China. Mycopathologia 2013; 175: 57–67. 22983901 10.1007/s11046-012-9577-0

[bib4] Capponi M, Segretain G, Sureau P. Penicillosis from *Rhizomys sinensis*. Bull Soc Pathol Exot Filiales 1956; 49: 418–421. 13364636

[bib5] Deng ZL, Yun M, Ajello L. Human *penicilliosis marneffei* and its relation to the bamboo rat (*Rhizomys pruinosus*. J Med Vet Mycol 1986; 24: 383–389. 3783360 10.1080/02681218680000581

[bib6] Chariyalertsak S, Vanittanakom P, Nelson KE et al. *Rhizomys sumatrensis* and *Cannomys badius*, new natural animal hosts of *Penicillium marneffei*. J Med Vet Mycol 1996; 34: 105–110. 8732355

[bib7] Gugnani H, Fisher MC, Paliwal-Johsi A et al. Role of *Cannomys badius* as a natural animal host of *Penicillium marneffei* in India. J Clin Microbiol 2004; 42: 5070–5075. 15528698 10.1128/JCM.42.11.5070-5075.2004 PMC525236

[bib8] Duong TA. Infection due to *Penicillium marneffei*, an emerging pathogen: review of 155 reported cases. Clin Infect Dis 1996; 23: 125–130. 8816141 10.1093/clinids/23.1.125

[bib9] Yuen KY, Wong SS, Tsang DN et al. Serodiagnosis of *Penicillium marneffei* infection. Lancet 1994; 344: 444–445. 7914566 10.1016/s0140-6736(94)91771-x

[bib10] Lee PP, Chan KW, Lee TL et al. Penicilliosis in children without HIV infection—are they immunodeficient? Clin Infect Dis 2012; 54: e8–e19. 22065867 10.1093/cid/cir754

[bib11] Segretain G. *Penicillium marneffei* n.sp., agent of a mycosis of the reticuloendothelial system. Mycopathologia 1959; 11: 327–353. 14444578 10.1007/BF02089507

[bib12] Di Salvo AF, Fickling AM, Ajello L. Infection caused by *Penicillium marneffei*: description of first natural infection in man. Am J Clin Pathol 1973; 60: 259–263. 4720403 10.1093/ajcp/60.2.259

[bib13] Pautler KB, Padhye AA, Ajello L. Imported *penicilliosis marneffei* in the United States: report of a second human infection. Sabouraudia 1984; 22: 433–438. 6505916 10.1080/00362178485380691

[bib14] Jayanetra P, Nitiyanant P, Ajello L et al. *Penicilliosis marneffei* in Thailand: report of five human cases. Am J Trop Med Hyg 1984; 33: 637–644. 6476209 10.4269/ajtmh.1984.33.637

[bib15] Deng ZL, Connor DH. Progressive disseminated penicilliosis caused by *Penicillium marneffei*. Report of eight cases and differentiation of the causative organism from *Histoplasma capsulatum*. Am J Clin Pathol 1985; 84: 323–327. 4036861 10.1093/ajcp/84.3.323

[bib16] Chan JK, Tsang DN, Wong DK. *Penicillium marneffei* in bronchoalveolar lavage fluid. Acta cytologica 1989; 33: 523–526. 2787573

[bib17] Chan YF, Woo KC. *Penicillium marneffei* osteomyelitis. J Bone Joint Surg Br 1990; 72: 500–503. 2341456 10.1302/0301-620X.72B3.2341456

[bib18] So SY, Chau PY, Jones BM et al. A case of invasive penicilliosis in Hong Kong with immunologic evaluation. Am Rev Respir Dis 1985; 131: 662–665. 3873195 10.1164/arrd.1985.131.4.662

[bib19] Yuen WC, Chan YF, Loke SL et al. Chronic lymphadenopathy caused by *Penicillium marneffei*: a condition mimicking tuberculous lymphadenopathy. Br J Surg 1986; 73: 1007–1008. 3790946 10.1002/bjs.1800731224

[bib20] Li JS, Pan LQ, Deng ZL et al. [A case report on *Penicillium marneffei*.]. J Clin Dermatol (China) 1985; 14: 24–26.

[bib21] Wang IL, Yeh HP, Chang SC et al. Penicilliosis caused by *Penicillium marneffei*. A case report. Derm Sin 1989; 7: 19–22.

[bib22] Chan YF, Chow TC. Ultrastructural observations on *Penicillium marneffei* in natural human infection. Ultrastructur Pathol 1990; 14: 439–452. 10.3109/01913129009007223 2247907

[bib23] Lee PP, Mao H, Yang W et al. *Penicillium marneffei* infection and impaired IFN-gamma immunity in humans with autosomal-dominant gain-of-phosphorylation STAT1 mutations. J Allergy Clin Immunol 2014; 133: 894–896.e5. 24188975 10.1016/j.jaci.2013.08.051

[bib24] Tang BS, Chan JF, Chen M et al. Disseminated penicilliosis, recurrent bacteremic nontyphoidal salmonellosis, and burkholderiosis associated with acquired immunodeficiency due to autoantibody against gamma interferon. Clin Vaccine Immunol 2010; 17: 1132–1138. 20445006 10.1128/CVI.00053-10 PMC2897261

[bib25] Chan JF, Chan TS, Gill H et al. Disseminated infections with *Talaromyces marneffei* in Non-AIDS patients given monoclonal antibodies against CD20 and kinase inhibitors. Emerg Infect Dis 2015; 21: 1101–1106. 26079984 10.3201/eid2107.150138 PMC4816330

[bib26] Tse E, Leung RY, Kwong YL. Invasive fungal infections after obinutuzumab monotherapy for refractory chronic lymphocytic leukemia. Ann Hematol 2015; 94: 165–167. 24879095 10.1007/s00277-014-2120-2

[bib27] Sisto F, Miluzio A, Leopardi O et al. Differential cytokine pattern in the spleens and livers of BALB/c mice infected with *Penicillium marneffei*: protective role of gamma interferon. Infect Immun 2003; 71: 465–473. 12496197 10.1128/IAI.71.1.465-473.2003 PMC143270

[bib28] Kudeken N, Kawakami K, Kusano N et al. Cell-mediated immunity in host resistance against infection caused by *Penicillium marneffei*. J Med Vet Mycol 1996; 34: 371–378. 8971625 10.1080/02681219680000671

[bib29] Kudeken N, Kawakami K, Saito A. CD4+ T cell-mediated fatal hyperinflammatory reactions in mice infected with *Penicillium marneffei*. Clin Exp Immunol 1997; 107: 468–473. 9067519 10.1046/j.1365-2249.1997.d01-945.x

[bib30] Kudeken N, Kawakami K, Saito A. Different susceptibilities of yeasts and conidia of *Penicillium marneffei* to nitric oxide (NO)-mediated fungicidal activity of murine macrophages. Clin Exp Immunol 1998; 112: 287–293. 9649193 10.1046/j.1365-2249.1998.00565.x PMC1904956

[bib31] Rongrungruang Y, Levitz SM. Interactions of *Penicillium marneffei* with human leukocytes in vitro. Infect Immun 1999; 67: 4732–4736. 10456924 10.1128/iai.67.9.4732-4736.1999 PMC96802

[bib32] Kawila R, Chaiwarith R, Supparatpinyo K. Clinical and laboratory characteristics of *penicilliosis marneffei* among patients with and without HIV infection in Northern Thailand: a retrospective study. BMC Infect Dis 2013; 13: 464. 24094273 10.1186/1471-2334-13-464 PMC3851520

[bib33] Louthrenoo W, Thamprasert K, Sirisanthana T. Osteoarticular penicilliosis marneffei. A report of eight cases and review of the literature. Br J Rheumatol 1994; 33: 1145–1150. 8000744 10.1093/rheumatology/33.12.1145

[bib34] Lo CY, Chan DT, Yuen KY et al. *Penicillium marneffei* infection in a patient with SLE. Lupus 1995; 4: 229–231. 7655496 10.1177/096120339500400313

[bib35] Lam KY, Cheung F, Yam LY et al. Atypical manifestations in a patient with systemic lupus erythematosus. J Clin Pathol 1997; 50: 174–176. 9155706 10.1136/jcp.50.2.174 PMC499750

[bib36] Hung CC, Hsueh PR, Chen MY et al. Invasive infection caused by *Penicillium marneffei*: an emerging pathogen in Taiwan. Clin Infect Dis 1998; 26: 202–203. 9455545 10.1086/517068

[bib37] Chim CS, Fong CY, Ma SK et al. Reactive hemophagocytic syndrome associated with *Penicillium marneffei* infection. Am J Med 1998; 104: 196–197. 9528739 10.1016/s0002-9343(97)00253-2

[bib38] Ko CI, Hung CC, Chen MY et al. Endoscopic diagnosis of intestinal *penicilliosis marneffei*: report of three cases and review of the literature. Gastrointest Endosc 1999; 50: 111–114. 10385737 10.1016/s0016-5107(99)70359-7

[bib39] Saadiah S, Jeffrey AH, Mohamed AL. *Penicillium marneffei* infection in a non aids patient: first case report from Malaysia. Med J Malaysia 1999; 54: 264–266. 10972040

[bib40] Pun TS, Fang D. A case of *Penicillium marneffei* osteomyelitis involving the axial skeleton. Hong Kong Med J 2000; 6: 231–233. 10895151

[bib41] Hsueh PR, Teng LJ, Hung CC et al. Molecular evidence for strain dissemination of *Penicillium marneffei*: an emerging pathogen in Taiwan. J Infect Dis 2000; 181: 1706–1712. 10823772 10.1086/315432

[bib42] Wong SS, Woo PC, Yuen KY. *Candida tropicalis* and *Penicillium marneffei* mixed fungaemia in a patient with Waldenström's macroglobulinaemia. Eur J Clin Microbiol Infect Dis 2001; 20: 132–135. 11305468 10.1007/pl00011243

[bib43] Wong SS, Wong KH, Hui WT et al. Differences in clinical and laboratory diagnostic characteristics of *penicilliosis marneffei* in human immunodeficiency virus (HIV)- and non-HIV-infected patients. J Clin Microbiol 2001; 39: 4535–4540. 11724878 10.1128/JCM.39.12.4535-4540.2001 PMC88582

[bib44] Wang JL, Hung CC, Chang SC et al. Disseminated *Penicillium marneffei* infection in a renal-transplant recipient successfully treated with liposomal amphotericin B. Transplantation 2003; 76: 1136–1137. 14557769 10.1097/01.TP.0000088667.02294.E7

[bib45] Liyan X, Changming L, Xianyi Z et al. Fifteen cases of penicilliosis in Guangdong, China. Mycopathologia 2004; 158: 151–155. 15518342 10.1023/b:myco.0000041842.90633.86

[bib46] Chan YH, Wong KM, Lee KC et al. Pneumonia and mesenteric lymphadenopathy caused by disseminated *Penicillium marneffei* infection in a cadaveric renal transplant recipient. Transpl Infect Dis 2004; 6: 28–32. 15225224 10.1111/j.1399-3062.2004.00038.x

[bib47] Woo PC, Lau SK, Lau CC et al. *Penicillium marneffei* fungaemia in an allogeneic bone marrow transplant recipient. Bone Marrow Transplant 2005; 35: 831–833. 15765113 10.1038/sj.bmt.1704895

[bib48] Deesomchok A, Tanprawate S. A 12-case series of *Penicillium marneffei* pneumonia. J Med Assoc Thai 2006; 89: 441–447. 16696387

[bib49] Beh CP, George J. Disseminated *Penicilium marneffei* infection. Med J Malaysia 2009; 64: 86–88. 19852332

[bib50] Ma BH, Ng CS, Lam R et al. Recurrent hemoptysis with *Penicillium marneffei* and *Stenotrophomonas maltophilia* in Job's syndrome. Can Respir J 2009; 16: e50–e52. 19707602 10.1155/2009/586919 PMC2734441

[bib51] Chan JF, Trendell-Smith NJ, Chan JC et al. Reactive and infective dermatoses associated with adult-onset immunodeficiency due to anti-interferon-gamma autoantibody: Sweet's syndrome and beyond. Dermatology 2013; 226: 157–166. 23652167 10.1159/000347112

[bib52] Lin JN, Lin HH, Lai CH et al. Renal transplant recipient infected with *Penicillium marneffei*. Lancet Infect Dis 2010; 10: 138. 20113983 10.1016/S1473-3099(10)70005-0

[bib53] Luo DQ, Chen MC, Liu JH et al. Disseminated *Penicillium marneffei* infection in an SLE patient: a case report and literature review. Mycopathologia 2011; 171: 191–196. 20842435 10.1007/s11046-010-9363-9

[bib54] Chong YB, Tan LP, Robinson S et al. Penicilliosis in lupus patients presenting with unresolved fever: a report of 2 cases and literature review. Trop Biomed 2012; 29: 270–276. 22735849

[bib55] Joosten SA, Hannan L, Heroit G et al. *Penicillium marneffei* presenting as an obstructing endobronchial lesion in an immunocompetent host. Eur Respir J 2012; 39: 1540–1543. 22654011 10.1183/09031936.00156911

[bib56] Hart J, Dyer JR, Clark BM et al. Travel-related disseminated *Penicillium marneffei* infection in a renal transplant patient. Transpl Infect Dis 2012; 14: 434–439. 22188555 10.1111/j.1399-3062.2011.00700.x

[bib57] Lee WI, Huang JL, Wu TS et al. Patients with inhibitory and neutralizing auto-antibodies to interferon-gamma resemble the sporadic adult-onset phenotype of Mendelian Susceptibility to Mycobacterial Disease (MSMD) lacking Bacille Calmette-Guerin (BCG)-induced diseases. Immunobiology 2013; 218: 762–771. 23083630 10.1016/j.imbio.2012.08.281

[bib58] Qiu Y, Zhang J, Liu G et al. A case of Penicillium marneffei infection involving the main tracheal structure. BMC Infect Dis 2014; 14: 242. 24886249 10.1186/1471-2334-14-242 PMC4030576

[bib59] De Monte A, Risso K, Normand AC et al. Chronic pulmonary penicilliosis due to *Penicillium marneffei*: late presentation in a french traveler. J Travel Med 2014; 21: 292–294. 24816045 10.1111/jtm.12125

[bib60] Furusawa H, Miyazaki Y, Sonoda S et al. *Penicilliosis marneffei* complicated with interstitial pneumonia. Intern Med 2014; 53: 321–323. 24531088 10.2169/internalmedicine.53.1465

[bib61] Liu GN, Huang JS, Zhong XN et al. *Penicillium marneffei* infection within an osteolytic lesion in an HIV-negative patient. Int J Infect Dis 2014; 23: 1–3. 24657269 10.1016/j.ijid.2013.12.019

[bib62] Xia XJ, Shen H, Xu AE. Cutaneous *Penicillium marneffei* infection in a patient with idiopathic CD4(+) lymphocytopenia. J Dermatol 2015; 42: 812–814. 25912013 10.1111/1346-8138.12899

[bib63] Jiang X, Zhou D. Diagnosis of *Penicillium marneffei* infection from a blood film. Br J Haematol 2015; 171: 670. 26250957 10.1111/bjh.13629

[bib64] Ye F, Luo Q, Zhou Y et al. Disseminated *penicilliosis marneffei* in immunocompetent patients: a report of two cases. Indian J Med Microbiol 2015; 33: 161–165. 10.4103/0255-0857.148433 25560026

[bib65] Wang PH, Wang HC, Liao CH. Disseminated *Penicillium marneffei* mimicking paradoxical response and relapse in a non-HIV patient with pulmonary tuberculosis. J Chin Med Assoc 2015; 78: 258–260. 25823679 10.1016/j.jcma.2013.11.009

[bib66] Stathakis A, Lim KP, Boan P et al. *Penicillium marneffei* infection in a lung transplant recipient. Transpl Infect Dis 2015; 17: 429–434. 25809145 10.1111/tid.12377

[bib67] Qiu Y, Zhang J, Liu G et al. Retrospective analysis of 14 cases of disseminated *Penicillium marneffei* infection with osteolytic lesions. BMC Infect Dis 2015; 15: 47. 25656710 10.1186/s12879-015-0782-6 PMC4322545

[bib68] Hoflich C, Sabat R, Rosseau S et al. Naturally occurring anti-IFN-gamma autoantibody and severe infections with *Mycobacterium cheloneae* and *Burkholderia cocovenenans*. Blood 2004; 103: 673–375. 12947000 10.1182/blood-2003-04-1065

[bib69] Doffinger R, Helbert MR, Barcenas-Morales G et al. Autoantibodies to interferon-gamma in a patient with selective susceptibility to mycobacterial infection and organ-specific autoimmunity. Clin Infect Dis 2004; 38: e10–e14. 14679469 10.1086/380453

[bib70] Browne SK, Burbelo PD, Chetchotisakd P et al. Adult-onset immunodeficiency in Thailand and Taiwan. N Engl J Med 2012; 367: 725–734. 22913682 10.1056/NEJMoa1111160 PMC4190026

[bib71] Chan JF, Yee KS, Tang BS et al. Adult-onset immunodeficiency due to anti-interferon-gamma autoantibody in mainland Chinese. Chin Med J (Engl) 2014; 127: 1189–1190. 24622460

[bib72] Chi CY, Chu CC, Liu JP et al. Anti-IFN-gamma autoantibodies in adults with disseminated nontuberculous mycobacterial infections are associated with HLA-DRB1*16:02 and HLA-DQB1*05:02 and the reactivation of latent varicella-zoster virus infection. Blood 2013; 121: 1357–1366. 23243276 10.1182/blood-2012-08-452482

[bib73] Tanaka Y, Hori T, Ito K et al. Disseminated *Mycobacterium avium* complex infection in a patient with autoantibody to interferon-gamma. Intern Med 2007; 46: 1005–1009. 17603241 10.2169/internalmedicine.46.6452

[bib74] Koya T, Tsubata C, Kagamu H et al. Anti-interferon-gamma autoantibody in a patient with disseminated *Mycobacterium avium* complex. J Infect Chemother 2009; 15: 118–122. 19396523 10.1007/s10156-008-0662-8

[bib75] Patel SY, Ding L, Brown MR et al. Anti-IFN-gamma autoantibodies in disseminated nontuberculous mycobacterial infections. J Immunol 2005; 175: 4769–4776. 16177125 10.4049/jimmunol.175.7.4769

[bib76] Ku CL, Lin CH, Chang SW et al. Anti-IFN-γ autoantibodies are strongly associated with HLA-DR*15:02/16:02 and HLA-DQ*05:01/05:02 across Southeast Asia. J Allergy Clin Immunol 2015pii: S0091-6749(15)01356-1. 10.1016/j.jaci.2015.09.018 26522403

[bib77] Kampmann B, Hemingway C, Stephens A et al. Acquired predisposition to mycobacterial disease due to autoantibodies to IFN-gamma. J Clin Invest 2005; 115: 2480–2488. 16127458 10.1172/JCI19316 PMC1190367

[bib78] Browne SK, Zaman R, Sampaio EP et al. Anti-CD20 (rituximab) therapy for anti-IFN-gamma autoantibody-associated nontuberculous mycobacterial infection. Blood 2012; 119: 3933–3939. 22403254 10.1182/blood-2011-12-395707 PMC3350360

[bib79] Czaja CA, Merkel PA, Chan ED et al. Rituximab as successful adjunct treatment in a patient with disseminated nontuberculous mycobacterial infection due to acquired anti-interferon-gamma autoantibody. Clin Infect Dis 2014; 58: e115–e118. 24336756 10.1093/cid/cit809 PMC3935498

[bib80] Zhou F, Bi X, Zou X et al. Retrospective analysis of 15 cases of Penicilliosis marneffei in a southern China hospital. Mycopathologia 2014; 177: 271–279. 24715630 10.1007/s11046-014-9737-5

[bib81] Ng WL, Chu CM, Wu AK et al. Lymphopenia at presentation is associated with increased risk of infections in patients with systemic lupus erythematosus. QJM 2006; 99: 37–47. 16371405 10.1093/qjmed/hci155

[bib82] Merayo-Chalico J, Gomez-Martin D, Pineirua-Menendez A et al. Lymphopenia as risk factor for development of severe infections in patients with systemic lupus erythematosus: a case-control study. QJM 2013; 106: 451–457. 23458779 10.1093/qjmed/hct046

[bib83] Wu TC, Chan JW, Ng CK et al. Clinical presentations and outcomes of *Penicillium marneffei* infections: a series from 1994 to 2004. Hong Kong Med J 2008; 14: 103–109. 18382016

[bib84] Seo JY, Ma YE, Lee JH et al. A case of disseminated *Penicillium marneffei* infection in a liver transplant recipient. Korean J Lab Med 2010; 30: 400–405. 20805713 10.3343/kjlm.2010.30.4.400

[bib85] Tong MK. Overview of Renal Transplant. Renal Transplantation in Hong Kong. Hong Kong Society of Transplantation: Hong Kong. 2006 Available athttp://www.hkst.org/the-education-corner/49-overview-of-renal-transplant.html/ accessed on 8 October 2015.

[bib86] Lie AK, Au WY, Liang R. Haematopoietic stem cell transplantation in Hong Kong. Hong Kong Med J 2009; 15 (3 Suppl 3): 17–21. 19494391

[bib87] Chan SC, Cheung TT, Chan AC et al. New insights after the first 1000 liver transplantations at The University of Hong Kong. Asian J Surg 2015 pii S1015-9584(15)00065-2. 10.1016/j.asjsur.2015.03.018 26143970

[bib88] Wong CF, Fung SL, Yan SW et al. Lung transplantation in Hong Kong: 12 years of experience. Respirology 2008; 13: 903–907. 18811889 10.1111/j.1440-1843.2008.01360.x

[bib89] Yuen KY, Woo PC, Ip MS et al. Stage-specific manifestation of mold infections in bone marrow transplant recipients: risk factors and clinical significance of positive concentrated smears. Clin Infect Dis 1997; 25: 37–42. 9243031 10.1086/514492

[bib90] Chim CS, Ho PL, Yuen ST et al. Fungal endocarditis in bone marrow transplantation: case report and review of literature. J Infect 1998; 37: 287–291. 9892534 10.1016/s0163-4453(98)92169-7

[bib91] Ho PL, Yuen KY. Aspergillosis in bone marrow transplant recipients. Crit Rev Oncol Hematol 2000; 34: 55–69. 10781748 10.1016/s1040-8428(00)00047-0

[bib92] Cheng VC, Chan JF, Ngan AH et al. Outbreak of intestinal infection due to *Rhizopus microsporus*. J Clin Microbiol 2009; 47: 2834–2843. 19641069 10.1128/JCM.00908-09 PMC2738128

[bib93] Woo PC, Leung SY, To KK et al. Internal transcribed spacer region sequence heterogeneity in *Rhizopus microsporus*: implications for molecular diagnosis in clinical microbiology laboratories. J Clin Microbiol 2010; 48: 208–214. 19906897 10.1128/JCM.01750-09 PMC2812305

[bib94] Cao L, Chan CM, Lee C et al. MP1 encodes an abundant and highly antigenic cell wall mannoprotein in the pathogenic fungus *Penicillium marneffei*. Infect Immun 1998; 66: 966–973. 9488383 10.1128/iai.66.3.966-973.1998 PMC108003

[bib95] Wong LP, Woo PC, Wu AY et al. DNA immunization using a secreted cell wall antigen Mp1p is protective against *Penicillium marneffei* infection. Vaccine 2002; 20: 2878–2886. 12126898 10.1016/s0264-410x(02)00234-7

[bib96] Woo PC, Lam CW, Tam EW et al. First discovery of two polyketide synthase genes for mitorubrinic acid and mitorubrinol yellow pigment biosynthesis and implications in virulence of *Penicillium marneffei*. PLoS Negl Trop Dis 2012; 6: e1871. 23094121 10.1371/journal.pntd.0001871 PMC3475676

[bib97] Yuen KY, Pascal G, Wong SS et al. Exploring the *Penicillium marneffei* genome. Arch Microbiol 2003; 179: 339–353. 12640520 10.1007/s00203-003-0533-8

[bib98] Yang E, Wang G, Woo PC et al. Unraveling the molecular basis of temperature-dependent genetic regulation in *Penicillium marneffei*. Eukaryot Cell 2013; 12: 1214–1224. 23851338 10.1128/EC.00159-13 PMC3811563

[bib99] Yang E, Chow WN, Wang G et al. Signature gene expression reveals novel clues to the molecular mechanisms of dimorphic transition in *Penicillium marneffei*. PLoS Genet 2014; 10: e1004662. 25330172 10.1371/journal.pgen.1004662 PMC4199489

[bib100] Woo PC, Zhen H, Cai JJ et al. The mitochondrial genome of the thermal dimorphic fungus *Penicillium marneffei* is more closely related to those of molds than yeasts. FEBS Lett 2003; 555: 469–477. 14675758 10.1016/s0014-5793(03)01307-3

[bib101] Woo PC, Tam EW, Chong KT et al. High diversity of polyketide synthase genes and the melanin biosynthesis gene cluster in *Penicillium marneffei*. FEBS J 2010; 277: 3750–3758. 20718860 10.1111/j.1742-4658.2010.07776.x

[bib102] Woo PC, Lau SK, Liu B et al. Draft genome sequence of *Penicillium marneffei* strain PM1. Eukaryot Cell 2011; 10: 1740–1741. 22131218 10.1128/EC.05255-11 PMC3232717

[bib103] Woo PC, Lam CW, Tam EW et al. The biosynthetic pathway for a thousand-year-old natural food colorant and citrinin in *Penicillium marneffei*. Sci Rep 2014; 4: 6728. 25335861 10.1038/srep06728 PMC4205486

[bib104] Woo PC, Chong KT, Tse H et al. Genomic and experimental evidence for a potential sexual cycle in the pathogenic thermal dimorphic fungus *Penicillium marneffei*. FEBS Lett 2006; 580: 3409–3416. 16714021 10.1016/j.febslet.2006.05.014

[bib105] Nimmanee P, Woo PC, Vanittanakom P et al. Functional analysis of atfA gene to stress response in pathogenic thermal dimorphic fungus *Penicillium marneffei*. PLoS One 2014; 9: e111200. 25365258 10.1371/journal.pone.0111200 PMC4218842

[bib106] Nimmanee P, Woo PC, Kummasook A et al. Characterization of sakA gene from pathogenic dimorphic fungus *Penicillium marneffei*. Int J Med Microbiol 2015; 305: 65–74. 25466206 10.1016/j.ijmm.2014.11.003

[bib107] Lau SK, Tse H, Chan JS et al. Proteome profiling of the dimorphic fungus *Penicillium marneffei* extracellular proteins and identification of glyceraldehyde-3-phosphate dehydrogenase as an important adhesion factor for conidial attachment. FEBS J 2013; 280: 6613–6626. 24128375 10.1111/febs.12566

[bib108] Lau SK, Chow WN, Wong AY et al. Identification of microRNA-like RNAs in mycelial and yeast phases of the thermal dimorphic fungus *Penicillium marneffei*. PLoS Negl Trop Dis 2013; 7: e2398. 23991243 10.1371/journal.pntd.0002398 PMC3749987

[bib109] Anolik JH, Friedberg JW, Zheng B et al. B cell reconstitution after rituximab treatment of lymphoma recapitulates B cell ontogeny. Clin Immunol 2007; 122: 139–145. 17008130 10.1016/j.clim.2006.08.009

[bib110] Seto WK, Chan TS, Hwang YY et al. Hepatitis B reactivation in patients with previous hepatitis B virus exposure undergoing rituximab-containing chemotherapy for lymphoma: a prospective study. J Clin Oncol 2014; 32: 3736–3743. 25287829 10.1200/JCO.2014.56.7081

[bib111] van Oers MH. CD20 antibodies: type II to tango? Blood 2012; 119: 5061–5063. 22653951 10.1182/blood-2012-04-420711

[bib112] Naqvi K, Verstovsek S, Kantarjian H et al. A potential role of ruxolitinib in leukemia. Expert Opin Investig Drugs 2011; 20: 1159–1166. 10.1517/13543784.2011.589383 PMC4143907 21635221

[bib113] Tong LX, Jackson J, Kerstetter J et al. Reactivation of herpes simplex virus infection in a patient undergoing ruxolitinib treatment. J Am Acad 2014; 70: e59–e60. 10.1016/j.jaad.2013.09.035 24528917

[bib114] Caocci G, Murgia F, Podda L et al. Reactivation of hepatitis B virus infection following ruxolitinib treatment in a patient with myelofibrosis. Leukemia 2014; 28: 225–227. 23929216 10.1038/leu.2013.235

[bib115] Wysham NG, Sullivan DR, Allada G. An opportunistic infection associated with ruxolitinib, a novel janus kinase 1,2 inhibitor. Chest 2013; 143: 1478–1479. 23648912 10.1378/chest.12-1604 PMC5991580

[bib116] Hopman RK, Lawrence SJ, Oh ST. Disseminated tuberculosis associated with ruxolitinib. Leukemia 2014; 28: 1750–1751. 24625550 10.1038/leu.2014.104

[bib117] Houben R, Voigt H, Noelke C et al. MAPK-independent impairment of T-cell responses by the multikinase inhibitor sorafenib. Mol Cancer Ther 2009; 8: 433–440. 19190114 10.1158/1535-7163.MCT-08-1051

[bib118] Zhao W, Gu YH, Song R et al. Sorafenib inhibits activation of human peripheral blood T cells by targeting LCK phosphorylation. Leukemia 2008; 22: 1226–1233. 18337760 10.1038/leu.2008.58

[bib119] Hipp MM, Hilf N, Walter S et al. Sorafenib, but not sunitinib, affects function of dendritic cells and induction of primary immune responses. Blood 2008; 111: 5610–5620. 18310500 10.1182/blood-2007-02-075945

[bib120] Teo M, O'Connor TM, O'Reilly SP et al. Sorafenib-induced tuberculosis reactivation. Onkologie 2012; 35: 514–516. 23007150 10.1159/000341829

[bib121] Wang YF, Xu HF, Han ZG et al. Serological surveillance for *Penicillium marneffei* infection in HIV-infected patients during 2004-2011 in Guangzhou, China. Clin Microbiol Infect 2015; 21: 484–489. 25677258 10.1016/j.cmi.2014.12.014

[bib122] Jan IS, Chung PF, Wang JY et al. Cytological diagnosis of *Penicillium marneffei* infection. J Formos Med Assoc 2008; 107: 443–447. 18583214 10.1016/S0929-6646(08)60151-5

[bib123] Woo PC, Lau CC, Chong KT et al. MP1 homologue-based multilocus sequence system for typing the pathogenic fungus *Penicillium marneffei*: a novel approach using lineage-specific genes. J Clin Microbiol 2007; 45: 3647–3654. 17881546 10.1128/JCM.00619-07 PMC2168507

[bib124] Wang YF, Cai JP, Wang YD et al. Immunoassays based on *Penicillium marneffei* Mp1p derived from Pichia pastoris expression system for diagnosis of penicilliosis. PLoS One 2011; 6: e28796. 22205971 10.1371/journal.pone.0028796 PMC3244411

[bib125] Cao L, Chen DL, Lee C et al. Detection of specific antibodies to an antigenic mannoprotein for diagnosis of *Penicillium marneffei* penicilliosis. J Clin Microbiol 1998; 36: 3028–3031. 9738061 10.1128/jcm.36.10.3028-3031.1998 PMC105105

[bib126] Yuen KY, Woo PC, Lau SK. A multilocus sequence typing system for Penicillium marneffei: an international molecular cyber system for tracking its origin and transmission. Hong Kong Med J 2010; 16: 45–46. 20864749

